# The Clinical Performance of the BioCode Respiratory Pathogen Panel for the Detection of Viruses and Bacteria from Nasopharyngeal Swabs

**DOI:** 10.1128/spectrum.04044-22

**Published:** 2023-04-11

**Authors:** Xin Zhang, Colleen Knoth, Anh Pham, Amorce Lima, Rosario Dominguez, Irvin Ibarra-Flores, Juan C. Lopez, Dominic Uy, Suzane Silbert, Anami Patel, Michael Aye, Yi-Wei Tang, Jennifer Dien Bard

**Affiliations:** a Memorial Sloan Kettering Cancer Center, New York, New York, USA; b Sichuan Academy of Medicine & Sichuan Provincial People’s Hospital, Chengdu, China; c Applied BioCode Inc., Santa Fe Springs, California, USA; d Tampa General Hospital, Tampa, Florida, USA; e Department of Pathology and Laboratory Medicine, Children’s Hospital Los Angeles, Los Angeles, California, USA; f Keck School of Medicine, University of Southern California, Los Angeles, California, USA; g PathAI Diagnostics, Memphis, Tennessee, USA; University of Maryland School of Medicine

**Keywords:** respiratory pathogens, syndromic testing, multiplex PCR, scalable throughput

## Abstract

Early detection of microbial pathogens causing respiratory tract infection plays a crucial role in clinical management. The BioCode Respiratory Pathogen Panel (BioCode RPP) utilizes reverse transcriptase PCR (RT-PCR) in combination with barcoded magnetic beads to amplify, detect, and identify respiratory pathogens. This panel qualitatively detects and identifies 14 viruses, including influenza virus A with H1 pdm09, H1, and H3 subtyping; influenza B; respiratory syncytial virus (RSV); human metapneumovirus; parainfluenza virus 1; parainfluenza virus 2; parainfluenza virus 3; parainfluenza virus 4; coronavirus (229E, NL63, OC43, and HKU1); adenovirus; and human rhinovirus/enterovirus, and 3 bacteria, including Chlamydia pneumoniae, Mycoplasma pneumoniae, and Bordetella pertussis. Reproducibility, which was assessed with contrived specimens containing 12 targets at 3 clinical sites, with 2 operators at each site for 5 days, was 99.4% for Flu A H3 and Flu B, 98.9% for RSV, and 100% for the remaining 9 targets assayed. A multicenter clinical trial evaluated the performance of the BioCode RPP with 2,647 nasopharyngeal swab specimens from 5 geographically distinct sites and revealed comparable performance between the BioCode RPP and FilmArray Respiratory Panel (FA-RP). Specifically, the positive percent agreements (PPAs) for various pathogens ranged between 80.8% and 100% compared with the FA-RP (1.7 and 2.0). Negative percent agreement ranged from 98.4% to 100% for BioCode RPP. The BioCode RPP also offers scalable automated testing capability of up to 96 specimens in a single run with total sample-to-result time under 5 h. The invalid rate of the BioCode RPP on initial testing was 1.0% (26/2,649).

**IMPORTANCE** Early detection of microbial pathogens causing respiratory tract infection plays a crucial role in clinical management. The BioCode Respiratory Pathogen Panel (BioCode RPP) is a high-throughput test that utilizes RT-PCR in combination with barcoded magnetic beads to amplify, detect, and identify 17 respiratory pathogens, including 14 viruses and 3 bacteria. This study summarizes data generated from a multicenter clinical trial evaluating the performance of the BioCode RPP on 2,647 nasopharyngeal swab specimens from five geographically distinct sites.

## INTRODUCTION

Upper respiratory tract infections (URTI) with similar signs and symptoms are caused by a wide variety of pathogens, and reliable diagnosis of URTI is important for the implementation of appropriate therapy, quarantine measures, and antibiotic stewardship. Molecular tests are considered the gold standard for the diagnosis of URTI, and a variety of assays that cater to small and large volume laboratories are currently cleared by the Food and Drug Administration (FDA) for *in vitro* diagnostic use (1). Among the various diagnostic tests available, multiplex nucleic acid amplification tests (NAATs) can simultaneously detect and identify a broad range of respiratory pathogens in a single test.

Depending on the clinical need and specimen volume, clinical laboratories may opt for a range of moderate to high-complexity molecular respiratory panels (1). As respiratory testing volumes can vary dramatically due to seasonality and viruses circulating in any given year, a scalable throughput system that can quickly accommodate these changing demands may be valuable. To address this need, the BioCode Respiratory Pathogen Panel (BioCode RPP) utilized reverse transcriptase PCR (RT-PCR) and barcoded magnetic beads (BMBs) to detect and identify respiratory pathogens using an automated platform (BioCode MDx-3000). It simultaneously detects and identifies 14 viruses, including influenza virus A (Flu A) with subtypes of H1 pdm09, H1, and H3; influenza B (Flu B); respiratory syncytial virus (RSV); human metapneumovirus (HMPV); parainfluenza virus 1 (PIV 1); parainfluenza virus 2 (PIV 2); parainfluenza virus 3 (PIV 3); parainfluenza virus 4 (PIV 4); human rhinovirus/enterovirus (RV/EV); adenovirus (ADV); and coronavirus (CoV) 229E, NL63, OC43, and HKU1, and 3 bacteria, including Chlamydia pneumoniae, Mycoplasma pneumoniae, and Bordetella pertussis, in a 96-well plate format.

Reported here are the results of a multicenter study to evaluate the clinical performance of the BioCode RPP compared with that of the FilmArray RP1.7 or RP2 assay. In addition, assay reproducibility was assessed with a panel of contrived samples at three sites with two operators at each site over 5 days.

## RESULTS

### Demographics.

**FIG 1:**
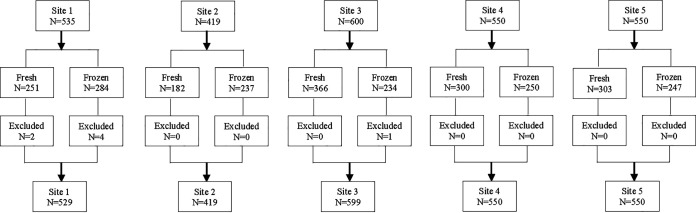
Distribution of specimens collected, tested, and reported for the prospective clinical study. NPS specimens were collected from 5 geographically distinct locations across the United States during the 2017 to 2019 respiratory seasons. The majority of the specimens were tested fresh, while some were collected and frozen prior to testing. Seven specimens did not meet the enrollment criteria and were excluded from the study.

A total of 2,654 prospective and retrospective nasopharyngeal swab (NPS) specimens were acquired for the clinical study; of these samples, 7 were excluded because the specimens did not meet the inclusion criteria after enrollment (e.g., insufficient volume, incomplete demographic data, or no data were obtained due to an invalid BioCode RPP test) (Fig. 1). Thus, a total of 2,647 specimens that met enrollment criteria and tested successfully were included in the final analysis. A total of 1,400 (52.9%) specimens were tested fresh and 1,247 (47.1%) had been frozen prior to testing on BioCode RPP. Sex and age were collected for all enrolled patients. There were nearly equal numbers of males (1,345 [50.8%]) and females (1,302 [49.2%]) enrolled. The age distribution included 1,034 (39.1%) patients 22 years of age or older and 1,613 (60.9%) patients of less than 22 years of age. A summary of patient demographic information is shown in Table 1.

**TABLE 1:** Demographic information and specimen processing for this prospective clinical study

Parameter	Data (*n* [%]) by:					
	Site 1	Site 2	Site 3	Site 4	Site 5	Total
Age category						
0–5 yrs	18 (3.4)	161 (38.4)	91 (15.2)	360 (65.5)	374 (68.0)	1,004 (37.9)
6–21 yrs	32 (6.0)	172 (41.1)	45 (7.5)	187 (34.0)	173 (31.5)	609 (23.0)
22–59 yrs	190 (35.9)	65 (15.5)	271 (45.2)	3 (0.5)	2 (0.4)	531 (20.1)
60+ yrs	289 (54.7)	21 (5.0)	192 (32.1)	0 (0)	1 (0.2)	503 (19.0)
Sex						
Male	271 (51.2)	195 (46.5)	265 (44.2)	320 (58.2)	294 (53.5)	1,345 (50.8)
Female	258 (48.8)	224 (53.5)	334 (55.8)	230 (41.8)	256 (46.5)	1,302 (49.2)
DNA extraction method						
easyMAG	529 (100%)	N/A^*a*^	599 (100%)	550 (100%)	N/A	1,678 (63.4)
MagNA Pure 96	N/A	419 (100%)	N/A	N/A	550 (100%)	969 (36.6)
Specimen type						
Fresh	249 (47.1)	182 (43.4)	366 (61.1)	300 (54.5)	303 (55.1)	1,400 (52.9)
Frozen	280 (52.9)	237 (56.6)	233 (38.9)	250 (45.5)	247 (44.9)	1,247 (47.1)

a N/A, not available.

### BioCode RPP performance.

BioCode RPP testing gave valid results on the first attempt for 2,623 of 2,649 specimens, representing a success rate of 99.0%. Twenty-four of 26 specimens were resolved after repeat testing. The final data set consisted of 2,647 specimens as described above. Positive percent agreements (PPAs) and negative percent agreements (NPAs) were calculated with respect to the comparator method (FilmArray Respiratory Panel [FA-RP]) for each pathogen. One analyte, namely, Flu A H1, was not detected by BioCode RPP or FA-RP during the trial, and therefore, no PPA or confidence interval (95% CI) could be calculated. The BioCode RPP demonstrated a PPA of ≥93.6% for 10 of the analytes and 100% for 3 analytes, namely, B. pertussis (2/2), C. pneumoniae (4/4), and M. pneumoniae (18/18). Due to the low number of positives for many analytes, some of the targets had a lower bound of the 95% CI below 80%. The NPA of the BioCode RPP was 98.4% or greater for all analytes. The lower boundaries of the 95% CI for NPA were 97.8% or greater for all targets. The summary of performance characteristics for individual BioCode RPP targets is presented in Table 2.

**TABLE 2:** Method comparison for BioCode RPP compared with FA-RP results before and after discordant analysis^*a*^

Pathogen	Initial results without discordant analysis						Adjudicated results after discordant analysis^*b*^					
	TP	TN	FN	FP	PPA (%)	NPA (%)	TP	TN	FN	FP	PPA (%)	NPA (%)
Adenovirus	68	2,528	10	41	87.2	98.4	94	2,534	4	15	95.9	99.4
Bordetella pertussis	2	2,626	0	19	100.0	99.3	16	2,626	0	5	100	99.8
Chlamydia pneumoniae	4	2,642	0	1	100.0	100.0	5	2,642	0	0	100	100
Coronavirus	111	2,492	22	22	83.5	99.1	123	2,506	8	10	93.9	100
Human metapneumovirus	135	2,488	7	17	95.1	99.3	144	2,491	4	8	97.3	99.7
Human rhinovirus/enterovirus	383	2,139	91	34	80.8	98.4	400	2,173	57	17	87.5	99.2
Influenza A	213	2,396	8	25	96.4	99.0	224	2,403	1	14	99.6	99.4
Influenza A H1	0	2,640	0	0	N/A	100.0	0	2,640	0	0	N/A	100
Influenza A H1 pdm09	52	2,578	1	9	98.1	99.7	61	2,578	1	0	98.4	100
Influenza A H3	147	2,474	10	9	93.6	99.6	152	2,481	3	4	98.1	99.8
Influenza B	51	2,579	3	14	94.4	99.5	56	2,581	1	9	98.2	99.7
Mycoplasma pneumoniae	18	2,609	0	20	100.0	99.2	31	2,609	0	7	100	99.7
Parainfluenza virus 1	15	2,630	2	0	88.2	100.0	15	2,632	0	0	100	100
Parainfluenza virus 2	10	2,632	2	3	83.3	99.9	11	2,635	1	0	91.7	100
Parainfluenza virus 3	118	2,508	4	17	96.7	99.3	130	2,509	3	5	97.7	99.8
Parainfluenza virus 4	16	2,627	2	2	88.9	99.9	16	2,629	2	0	88.9	100
Respiratory syncytial virus	200	2,422	4	21	98.0	99.1	204	2,426	0	17	100	99.3

a Results are no. of specimens unless otherwise indicated. TP, true positive; TN, true negative; FP, false positive; FN, false negative; PPA, positive percent agreement; NPA, negative percent agreement.

b Discordant analysis was performed with validated PCR/bidirectional sequencing and/or alternative NAAT and repeat testing with BioCode RPP depending on the availability of residual specimens.

Compared with the FA-RP, the BioCode RPP had an overall PPA of 85.9% (1,543/1,797) and NPA of 99.6% (43,010/43,176). Discordant analysis was performed on all false-positive (FP) and false-negative (FN) results for samples (249 and 162 tests, respectively), except for 1 sample that had an insufficient volume. The discordant analysis supported the BioCode RPP results for 143 positive tests (57%, 143/249) and 81 negative tests (50%, 81/162). The adjusted overall PPA is 95.2% (1,682/1,767) and NPA is 99.7% (43,095/43,206). The individual PPAs are ≥91.7% for 14 of the RPP the analytes after discordant analysis.

The two analytes with a PPA of <90.0% after discordant analysis were RV/EV (87.5%, 400/457) and PIV 4 (88.9%, 16/18). The PPA for Flu A H1 could not be calculated due to a lack of positive clinical specimens. The NPA for each analyte of the BioCode RPP after discordant analysis was ≥99.2%. The summary of the adjudicated results with discordant analysis is presented in Table 2. The following are some noteworthy findings after discordant analysis.

Adenovirus PPA increased from 87.2% (68/78) to 95.9% (94/98). Six of the 10 FN results could not be confirmed by PCR/bidirectional sequencing or an alternative NAAT. In addition, discordant analysis supported the addition of 26 samples detected by BioCode RPP and not detected by the comparator on initial analysis (22 FA-RP and 4 FA-RP2).

Coronavirus PPA increased from 83.5% (111/133) to 93.9% (123/130). Of the 22 FNs, 14 could not be confirmed by PCR/bidirectional sequencing or an alternative NAAT. Also, discordant analysis supported the addition of 12 of the 22 FP specimens as true positives, which were not detected by FA-RP. Of those specimens, 8 were identified as CoV OC43 and 4 as CoV NL63 by sequencing.

Human RV/EV PPA increased from 80.8% (383/474) to 87.5% (400/457). Of the 91 FNs, 34 could not be confirmed by PCR/bidirectional sequencing or an alternative NAAT. Twenty-eight of the 57 confirmed positive specimens were detected by PCR/bidirectional sequencing (20 RV C, 1 RV A, 2 EV-D68, and 5 coxsackievirus specimens), 25 were detected by an alternative NAAT, and 4 were detected by BioCode RPP upon repeat. Twelve of the 57 FNs after follow-up testing were detected by BioCode RPP repeat. Also, 17 of the 34 FPs were detected by follow-up testing.

Bordetella pertussis PPA was initially based on 2 specimens (100%, 2/2). The BioCode RPP detected 19 additional positives compared with FA-RP. Of these 19 specimens, 14 were supported by follow-up testing with adjudicated PPA of 100% (16/16). Similarly, while M. pneumoniae had a 100% PPA (18/18), after discordant analysis, the total adjudicated TP increased to 31 with 100% PPA.

Comparing the PPA of RSV, HMPV, and Flu virus subtyping by BioCode RPP in different respiratory seasons, we found that fresh specimens showed higher PPA for Flu B than that of frozen specimens from 2017 to 2018 (chi-square test, *P* = 0.0001), while there was no significant difference among other Flu subtypes, RSV, and HMPV (Table 3).

**TABLE 3:** RSV, HMPV, and influenza virus subtyping by BioCode RPP in different flu seasons

Pathogen	Subtyping results (% [*n*]) by specimen type and season			*P* value
	Fresh	Frozen		
	2019	2017–2018	2019	
Influenza A H1 pdm09	96.7 (29/30)	100 (10/10)	100 (13/13)	
Influenza A H3	93.2 (82/88)	97.0 (33/34)	91.4 (32/35)	
Influenza B	100 (7/7)	93.6 (44/47)	N/A^*a*^	0.0001^*b*^
Respiratory syncytial virus	97.8 (91/93)	97.9 (46/47)	98.4 (63/64)	
Human metapneumovirus	95.7 (89/93)	92.6 (25/27)	95.5 (21/22)	

a Flu B was not detected in any specimens frozen in 2019.

b PPA of Flu B frozen specimens (2017–2018) compared with that of fresh specimens, *P* < 0.05.

Despite that over 2,600 prospective specimens were enrolled, few or no positives were detected for some pathogens (CoV 229E, HKU1, PIV1, PIV2, PIV4, B. pertussis, C. pneumoniae, and M. pneumoniae) (Table 2). To evaluate the performance of BioCode RPP for those pathogens, the prospective collection was supplemented with previously characterized archived positive and negative specimens (Table 4). PPA was 100% for the bacterial targets and PIV 3. In addition, the PPA for archived positives was 12/13 (92.3%) for PIV 1, 19/20 (95%) for PIV 2, and 14/15 (93.3%) for PIV 4. The PPA for CoV HKU-1 was 37/40 (92.5%) and that for CoV 229E was only 14/18 (77.8%); 2 out of 4 initial false negatives were detected upon retest, indicating that those specimens may be borderline positives for RPP. Very few archived positives for C. pneumoniae and none for Flu A H1 were collected. Therefore, the test performance results for these pathogens were validated primarily with contrived specimens using various strains at multiple concentrations (data not shown).

**TABLE 4:** Results from archived specimens tested by the BioCode RPP with the easyMAG extraction system

Target	*n*	Positive agreement		Negative agreement	
		PA (*n* [%])	95% CI	NA (*n* [%])	95% CI
Bordetella pertussis	165	10/10 (100)	(69.2, 100)	144/155 (92.9)	(87.7, 96.4)
Chlamydia pneumoniae	165	10/10 (100)	(69.2, 100)	155/155 (100)	(97.6, 100)
Coronavirus 229E	165	14/18 (77.8)	(52.4, 93.6)	147/147 (100)	(97.5, 100)
Coronavirus HKU1	165	37/40 (92.5)	(79.6, 98.4)	118/125 (94.4)	(88.8, 97.7)
Mycoplasma pneumoniae	165	7/7 (100)	(59.0, 100)	153/158 (96.8)	(92.8, 99.0)
Parainfluenza virus 1	165	12/13 (92.3)	(64.0, 99.8)	152/152 (100)	(97.6, 100)
Parainfluenza virus 2	165	19/20 (95.0)	(75.1, 99.9)	144/145 (99.3)	(96.2, 100)
Parainfluenza virus 4	165	14/15 (93.3)	(68.1, 99.8)	150/150 (100)	(97.6, 100)

Collectively, RPP gave similar detection results for each pathogen with either extraction system. The limit of detection (LoD) was within 3-fold for each pathogen between easyMAG and MagNA Pure 96 systems (data not shown). Reproducibility was performed with both extraction systems with equivalent results (Table 5). Both extraction systems were cleared for use with BioCode RPP by the U.S. FDA.

**TABLE 5:** Reproducibility of BioCode RPP^*a*^

Pathogen	No. of samples detected/tested (% agreement) by site and test			Total no. of samples detected/tested (%) (95% CI)
	Site 1, easyMAG	Site 2, MagNA Pure	Site 3, easyMAG	
Adenovirus	60/60 (100)	60/60 (100)	60/60 (100)	180/180 (100) (97.9–100.0)
Coronavirus NL63	60/60 (100)	60/60 (100)	60/60 (100)	180/180 (100) (97.9–100.0)
Human metapneumovirus	60/60 (100)	60/60 (100)	60/60 (100)	180/180 (100) (97.9–100.0)
Human rhinovirus/enterovirus	60/60 (100)	60/60 (100)	60/60 (100)	180/180 (100) (97.9–100.0)
Influenza A H3^*b*^	59/60 (98.3)	60/60 (100)	60/60 (100)	179/180 (99.4) (96.9–99.9)
Influenza B^*b*^	59/60 (98.3)	60/60 (100)	60/60 (100)	179/180 (99.4) (96.9–99.9)
Parainfluenza virus 2	60/60 (100)	60/60 (100)	60/60 (100)	180/180 (100) (97.9–100.0)
Parainfluenza virus 3	60/60 (100)	60/60 (100)	60/60 (100)	180/180 (100) (97.9–100.0)
Respiratory syncytial virus^*b*^^,^^*c*^	58/60 (96.7)	60/60 (100)	60/60 (100)	178/180 (98.9) (96.0–99.7)
Bordetella pertussis	60/60 (100)	60/60 (100)	60/60 (100)	180/180 (100) (97.9–100.0)
Chlamydia pneumoniae	60/60 (100)	60/60 (100)	60/60 (100)	180/180 (100) (97.9–100.0)
Mycoplasma pneumoniae	60/60 (100)	60/60 (100)	60/60 (100)	180/180 (100) (97.9–100.0)

a Reproducibility was assessed with a panel of samples at 3 different sites, 2 with easyMAG and 1 with MagNA Pure 96 extraction systems.

b Low positive, 1.5 times LoD not detected.

c Medium positive, 3 times LoD not detected.

The reproducibility of the BioCode RPP was evaluated at 3 investigational sites (2 with the easyMAG system and 1 with the MagNA Pure96 system) and consisted of a 7-sample panel, including 1 negative and 6 positives, with each seeded with 4 organisms in a simulated NPS matrix (2 at 1.5 times the LoD and 2 at 3 times the LoD). With this design, the panel included 12 representative analytes; all analytes were detected by the BioCode RPP except for Flu A H1 pdm09, Flu A H1, PIV 1, and PIV 4. The panel was tested in triplicate over 5 days by 2 operators at each site. A total of 30 runs (10 per site), 630 samples, 2,160 positive results, and 8,000 negative results obtained during 5 days of testing were analyzed. The reproducibility study showed excellent within-site and site-to-site reproductivity (Table 5). The data showed a low rate of false positives (2 at site 3) and false negatives (2 at 1.5 times the LoD and 1 at 3 times the LoD; at site 1). Positive agreements across all sites were 100% for all but the following 2 targets: RSV (98.9%, 178/180) and Flu A H3 (99.4%, 179/180).

This prospective study also included subjects of various ages, ranging from a few months to 98 years. Among this cohort, a higher prevalence for certain viruses was observed in children of ≤5 years of age than that of patients of all ages; these viruses included RSV (15.5% versus 8.3%), HMPV (9.4% versus 5.7%), ADV (8.0% versus 4.1.%), PIV 3 (7.4% versus 5.1%), and RV/EV (23.3% versus 15.8%). In contrast, Flu A (both H3 and H1pdm subtypes) and Flu B were most prevalent in the age group 6 to 21 years at 35.3% and 40.0%, respectively. We also found that B. pertussis (57.1%) and M. pneumoniae (55.3%) were most common in the age group 6 to 21 years.

## DISCUSSION

**FIG 2:**
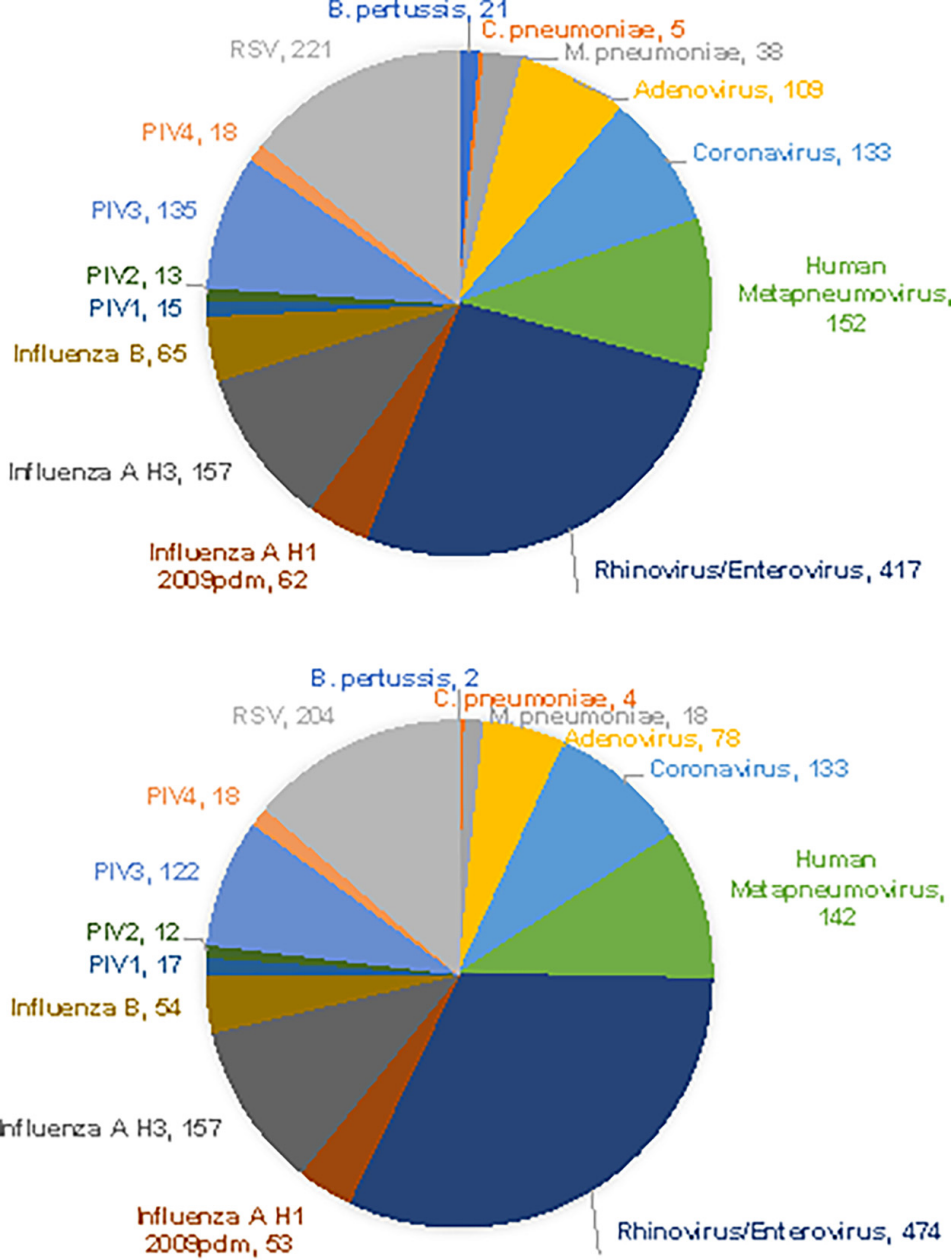
Prevalence of pathogens detected by BioCode RPP (top) and FA-RP (bottom) during the prospective clinical study.

Our study showed that the prevalence of pathogens in NPS samples during the clinical trial period of 2017 to 2018 and 2018 to 2019 respiratory seasons was detected similarly by BioCode RPP and FilmArray assays. Among the prospective specimens, the most frequently detected pathogen by BioCode RPP was RV/EV (*n* = 417), followed by RSV (*n* = 221), Flu A H3 (*n* = 157), HMPV (*n* = 152), PIV 3 (*n* = 135), CoV (*n* = 133), ADV (*n* = 109), Flu B (*n* = 65), and Flu A H1 pdm09 (*n* = 62). On the other hand, some pathogens (PIV 1, PIV 2, PIV 4, and C. pneumoniae) were detected in less than 20 specimens, and Flu A H1 was detected in none of the specimens. Despite some discordant results, the overall distribution of respiratory pathogens detected was similar between RPP and FilmArray panels (Fig. 2). The composition of pathogens is also similar to that reported for a medical center in the 2013 to 2014 season (2).

During respiratory seasons in North America prior to 2020, influenza viruses and RSV predominated as etiological agents in patients seeking health care, although other respiratory viruses were also detected. During the study period from 2017 to 2019, the Flu A H3 subtype was more common than the H1 pdm09 and influenza B. Between two respiratory seasons, more positively identified Flu A (both H3 and pdm09) specimens were detected from fresh specimens collected in 2019 than those from frozen specimens collected during 2017 to 2018 season and 2018 to 2019 season. A similar pattern was observed for HMPV and RSV. In contrast, more Flu B-positive specimens were detected from frozen specimens collected during 2017 to 2018 season than those from combined fresh and frozen specimens collected in 2019. This result reflects the higher prevalence of Flu B in 2017 to 2018 season which was classified as high severity by the CDC (3). The 2018-2019 influenza season was less severe compared to peak activity in 2017-2018. However, a protracted season due to distinct waves of Flu A H1pdm and H3N2 resulted in a similar burden of illness in children by the end of that season (4).

The overwhelming majority of the organisms detected by RPP were viral pathogens (1,733/1,797, 96.4%), whereas bacterial pathogens made up only 3.6% of total positives. This distribution is consistent with findings reported by Popowitch et al. (5, 6). Although negative results cannot rule out other bacterial infections, the detection of viruses in symptomatic patients may indicate against prescribing antibiotics (7). Positive identification of viruses by respiratory panels has been shown by other investigators to reduce unnecessary antibiotic use, thereby supporting antibiotic stewardship programs (8, 9).

RV/EV was the most frequently detected pathogen (23.2% of pathogens detected by the BioCode RPP), which is consistent with previous reports (5, 6, 10). Therefore, it is not surprising that the most common coinfection combinations include RV/EV detected alongside another target (see Table S1 in the supplemental material). The following pathogens were most frequently codetected from prospective specimens: RV/EV and RSV (17), RV/EV and ADV (13), RV/EV and HMPV (13), and RV/EV and influenza A (11). Among the prospective collection, 22 specimens showed codetection of 3 different pathogens, and 3 specimens had 4 or 5 pathogens codetected by RPP. The clinical significance of such codetection is unclear as the severity of infection was outside the scope of this study. Coinfections with certain viruses may be more significant. For instance, one study suggested that patients with a coinfection of Flu A H1pdm09 with RV appeared to have lower disease severity than patients with coinfection with other viruses (11). However, Choi et al. (12) observed that the codetection of multiple pathogens was associated with chronic lung disease rather than the severity of acute respiratory infections. Several studies on pediatric patients evaluated the effect of single infections or coinfections of respiratory viruses, such as RSV, and viral load on the severity of infection or antibiotic use (13–16).

Findings from our study revealed significant differences in the detection of certain targets between the two assays. Specifically, more cases of CoV-229E and HKU1 were detected by the FA-RP assay, and the results may be interpreted as the assay having higher sensitivity for these organisms. However, FPs cannot be ruled out, as several FA-RP-positive specimens were not confirmed by sequencing or an alternate FDA-cleared multiplex NAAT. On the other hand, considerably more positive results for bacterial pathogens, especially B. pertussis and M. pneumoniae, were reported by BioCode RPP than FA-RP. It is well documented that FilmArray (FA-RP and FA-RP2) uses a single copy target (*ptx*) for B. pertussis, compared with the BioCode RPP which uses the multicopy target (IS481). This difference may account for the lower sensitivity observed in this study as well as the higher reported LoD for FA-RP and FA-RP2 (4,000 CFU/mL and 1,000 CFU/mL, respectively) than that of BioCode RPP (15 CFU/mL) (17, 18). The detection of additional ADV positives by the BioCode RPP compared with FA-RP is in line with a higher LoD for FA-RP (300 50% tissue culture infective dose [TCID_50_]/mL) than that of RPP (18 TCID_50_/mL) for species C. In addition, while the BioCode RPP is designed to detect all ADV species A to F, previous studies demonstrated poor sensitivity for species A, D, and F with FilmArray RP1, prompting updated primers and additional assays in FA-RP2 (6).

Our study showed a significant difference in the detection of RV/EV between the two assays, and some FN results for RPP could not be confirmed by sequencing or another FDA-cleared assay. The analytical sensitivity of BioCode RPP was comparable to that of other FDA-cleared tests (data not shown); however, there are numerous subtypes for RV and EV, and sensitivity differences for particular subtypes may have contributed to the lower PPA. RV/EV and CoVs cause the common cold with mild respiratory symptoms but may associate with the hospitalization of older adults (19), and the detection of these viruses may be more pertinent in immunocompromised patients (20). It is also important to detect clinically important enteroviruses, including EV-D68, which have been reported to be associated with the development of acute flaccid myelitis (AFM) (21) and EV-A71 which can cause neurological disease (22). Although we were unable to obtain well-characterized specimens for these viruses, *in silico* analysis does not predict performance issues for these important subtypes.

The commercial NAAT respiratory panels represent remarkable progress in the laboratory diagnosis of upper respiratory tract infections. In this multicenter clinical study, the performance characteristics of the BioCode RPP on the BioCode MDx-3000 instrument were evaluated by comparison with the FilmArray RP, a well-known multiplex NAAT panel. Similar to the FA-RP, BioCode RPP provides results for a large panel of viral and bacterial agents for respiratory infections in a workflow format with reasonable turnaround time to results.

In contrast to the sample-to-answer approach, the BioCode MDx-3000 system offers solutions for laboratories that require batched testing and scalable throughput for significant sample volume with limited hands-on time. The automated process enables laboratory personnel to perform other tasks while the specimens are being processed or analyzed. However, it requires a longer time to results than cartridge-based systems that are convenient for a small number of specimens. Therefore, the BioCode system may be better suited for high-throughput laboratories that perform syndromic testing on a larger scale. In contrast to FA-RP, BioCode RPP enables clinical laboratories to report select pathogen results based on clinician request instead of reporting results on all pathogens, which can potentially create confusion or ethical dilemmas for health care professionals.

There are some limitations to this study. Although the study was performed in geographically distinct areas over two respiratory seasons in the United States and despite the enrollment of 2,647 prospective specimens, few or no positives were detected for some pathogens, including coronavirus types 229E and HKU1, PIV1, PIV2, PIV4, B. pertussis, C. pneumoniae, and M. pneumoniae. Therefore, we supplemented with previously characterized archived positive or contrived specimens. Over the course of the study, the standard of care comparator was updated from FA-RP to FA-RP2 at one site (see Table S2 in the supplemental material), which would further limit the number of positive samples; for this analysis, the results are combined for both comparator assays despite differences for several targets (23). A breakdown by each FA-RP version is available as supplemental data (see Table S3 in the supplemental material). Additional studies with rare positive clinical specimens are needed to better characterize the performance of BioCode RPP for such pathogens. Moreover, the impact of these multiplex panels on patient care was not evaluated in this study. Additional studies may yield clinically relevant information regarding the utility and impact of multiplex respiratory tests on the impact of patient care, such as the length of stay, antibiotic stewardship, and overall cost for patient care.

In this multicenter study, the BioCode RPP yielded highly accurate results for respiratory pathogens, including the detection of polymicrobial infections from NPS. The assay offers a potential solution for large-size clinical and public health laboratories that require high-throughput and scalable testing platforms for upper respiratory tract infections.

## MATERIALS AND METHODS

### Clinical specimens.

A total 2,654 remnant NPSs in viral or universal transport medium (VTM/UTM) were saved after standard of care testing (FilmArray RP or RP2; FA-RP) during the 2017 to 2019 respiratory seasons from 5 geographically diverse clinical sites (Memorial Sloan Kettering Cancer Center, New York, NY; Tampa General Hospital, Tampa, FL; Children’s Hospital of Los Angeles, Los Angeles, CA; Children’s Hospital of Colorado, Denver, CO; and Poplar Healthcare, Memphis, TN). Specimens were prospectively collected and either tested fresh with the BioCode RPP (2 to 8°C for no more than 7 days) or stored frozen (<–70°C) for up to 18 months before testing (Fig. 1). Clinical and demographic data were collected for each specimen, including age, sex, hospitalization status, FA-RP or FA-RP2 results, date of specimen collection, specimen storage status (fresh or frozen), and extraction method (for BioCode RPP only). The study was approved by the local institutional review boards (IRBs) at each of the clinical sites. Inclusion criteria for archived specimens were nasopharyngeal swabs in VTM or UTM stored at ≤–70°C with sufficient volume for PCR/bidirectional sequencing and BioCode RPP testing. Archived positive specimens were enrolled for coronavirus types 229E and HKU1, PIV1, PIV2, PIV4, B. pertussis, C. pneumoniae, and M. pneumoniae. Specimens were handled in a blind manner and mixed with negatives prior to shipping to clinical sites for testing with BioCode RPP and FA-RP.

### BioCode RPP testing.

Total nucleic acids were extracted from 200 μL of nasopharyngeal swab specimens in VTM spiked with 10 μL of BioCode RNA IC2 using the NucliSENS easyMAG (bioMérieux, Durham, NC) or MagNA Pure 96 (MP96; Roche Applied Science, Manheim, Germany) systems and then eluted in 50 μL elution buffer. The nucleic acid eluate (5 μL) for each sample was mixed with 20 μL BioCode RPP PCR reagents in a 96-well PCR plate and assayed on the BioCode MDx-3000 instrument. The BioCode MDx-3000 instrument automates multiplex reverse transcriptase PCR, target capture/labeling, and detection steps. The spiked BioCode RNA IC2 serves as an internal process control for each specimen and an external negative control to validate results for each plate. The BioCode MDx software provides automated result analysis for each target in a valid run; qualitative results of “detected,” “not detected,” or “indeterminate” (for Flu A only) are determined based on median fluorescence intensity (MFI) compared with target-specific threshold values. The BioCode RPP employs endpoint PCR, and MFI values are not proportional to the quantity of the viral or bacterial target present in the sample. If the internal control fails, the software automatically provides a result of “invalid” for all undetected panel analytes.

### Comparator methods.

Each clinical specimen was tested by the FilmArray RP1.7 or RP2 (FA-RP; BioFire Diagnostics, Salt Lake City, UT) according to the manufacturer’s instructions as described previously (24). In brief, 1 mL hydration solution was added to the pouch using a cannula. Using the transfer pipette provided in the test kit, approximately 300 μL of sample was added to the sample injection vial, and the consequent sample mixture was transferred to the pouch using a sample-loading cannula. The pouch was then put into the FilmArray instrument, and the test was performed and interpreted using the FilmArray operating software.

Discordant specimens were subjected to bidirectional PCR/sequencing. The assays utilized automated nucleic acid extraction (NucliSENS easyMAG system). Amplification and presumptive identification of positive result based on predetermined melting temperature (*T_m_*) ranges for each of the assays were achieved with real-time PCR with SYBR green using ABI 7500 system, followed by bidirectional sequencing with the ABI 3500 analyzer. The resulting sequencing data were analyzed with ABI sequence scanner software v.2 and SeqMan Pro (Lasergene 12 Core Suite) software to generate Phred scores and contig length and ambiguous nucleotides, respectively. A NCBI BLAST search of each of resulting contigs was performed to generate identity to reference, query coverage, and expected value (E value). The minimum requirements for acceptability were as follows: Phred score of ≥20, contig length of ≥200 bases, identity of ≥95%, and coverage of ≥95%.

All discordant specimens with sufficient volume were extracted with the NucliSENS easyMAG system (bioMérieux) for additional testing. Multiplex RT-PCR for NxTAG RPP and detection on a MagPix instrument (Luminex) were performed according to the package insert instructions.

### Data analysis.

Results generated by the BioCode RPP were compared with results from FA-RP1.7/2.0 for all analytes. Positive percent agreements (PPAs) and negative percent agreements (NPAs) were determined. PPAs were calculated as 100 × [TP/(TP + FN)], with TP, true positive or positive by both FA-RP and BioCode RPP; and FN, false negative or negative by BioCode RPP only. NPAs were calculated as 100 × [TN/(TN+ FP)]. The binomial two-sided 95% confidence intervals (CI) were calculated for both performance measures according to the Wilson score method. The chi-square test was used to assess PPA differences between fresh specimens and frozen specimens. *P* values were calculated, and values of <0.05 were considered statistically significant.

All discordant samples with sufficient volume were tested with validated bidirectional PCR/sequencing assays, repeated on the BioCode RPP, and tested with an alternative NAAT (false-negative result for CoV, ADV, or RV/EV only) assay that was cleared by the U.S. Food and Drug Administration (FDA) for *in vitro* diagnostic use. A composite algorithm was used to determine the correct call for false-positive (FP) specimens; detected results for two or more of the assays (FA-RP, BioCode RPP, or bidirectional PCR/sequencing) were considered true positive (TP). For false-negative (FN) specimens, all follow up tests (BioCode RPP repeat, bidirectional PCR/sequencing, and alternative FDA-cleared NAAT) must be not detected to be considered a TN. In addition, to monitor risks of contamination, all clinical sites were required to run external controls for reproducibility and method comparison studies, and results were monitored throughout the study period. A carry-over contamination study revealed minimal risks of contamination throughout the procedure.
