# Supplementary material for: The Clinical Performance of the BioCode Respiratory Pathogen Panel for the Detection of Viruses and Bacteria from Nasopharyngeal Swabs
Source: Microbiol Spectr. 2023 Apr 11;11(3):e04044-22. doi: 10.1128/spectrum.04044-22 (PMC10269715; doi:10.1128/spectrum.04044-22)
Supplement: Supplemental file 1 — Tables S1 to S3. Download spectrum.04044-22-s0001.pdf, PDF file, 0.2 MB [file spectrum.04044-22-s0001.pdf]

Supplemental Table 1. Most common co-infection combinations detected by BioCode RPP. Only co-infections with 5 or more instances are included in this table.

Co-Infection Combination		Number of Specimens
Organism A	Organism B	
Rhinovirus/Enterovirus	Respiratory Syncytial Virus	17
Rhinovirus/Enterovirus	Adenovirus	13
Rhinovirus/Enterovirus	Human Metapneumovirus	13
Rhinovirus/Enterovirus	Influenza A	11
Rhinovirus/Enterovirus	Parainfluenza Virus 3	10
Adenovirus	Respiratory Syncytial Virus	10
Rhinovirus/Enterovirus	*Bordetella pertussis*	6
Adenovirus	Parainfluenza Virus 3	5

Supplemental Table 2. Breakdown of specimens enrolled in prospective clinical study by site, storage, extraction method and comparator method.

Site	Samples enrolled	Storage		Extraction Method		FA-RP Version	
		Fresh	Frozen	easyMAG	MP 96	RP1	RP2
Site 1	530	259	271	530	0	277	253
Site 2	419	184	235	0	419	419	0
Site 3	600	374	226	600	0	0	600
Site 4	550	300	250	550	0	550	0
Site 5	550	303	247	0	550	550	0
**Total:**	**2649**	**1420**	**1229**	**1680**	**969**	**1796**	**853**

MP 96 = MagNA Pure 96, FA-RP = FilmArray Respiratory Panel (Comparator)

Supplemental Table 3. Comparison for BioCode RPP results compared to different versions of FilmArry RP before discordant analysis

Target	FilmArray RP1.7			FilmArray RP2		
	(n)	Positive Agreement PA (%)	Negative Agreement NA (%)	(n)	Positive Agreement PA (%)	Negative Agreement NA (%)
Adenovirus	1796	48/50 (96%)	1709/1746 (97.9%)	851	20/28 (71.4%)	819/823 (99.5%)
Bordetella pertussis	1796	2/2 (100%)	1776/1794 (99.0%)	851	N/A†	850/851 (99.9%)
Chlamydia pneumoniae	1796	4/4 (100%)	1791/1792 (99.9%)	851	N/A†	851/851 (100%)
Coronavirus	1796	93/101 (92.1%)	1677/1695 (98.9%)	851	22/37 (59.5%)	809/814 (99.4%)
Human Metapneumovirus	1796	103/106 (97.2%)	1674/1690 (99.1%)	851	32/36 (88.9%)	814/815 (99.9%)
Human Rhinovirus/Enterovirus	1796	293/359 (81.6%)	1408/1437 (98.0%)	851	90/115 (78.3%)	731/736 (99.3%)
Influenza A	1795	168/169 (99.4%)	1605/1626 (98.7%)	849	44/51 (86.3%)	795/798 (99.6%)
Influenza A H1	1796	N/A†	1796/1796 (100%)	848	N/A†	848/848 (100%)
Influenza A H1 2009pdm	1796	37/37 (100%)	1750/1759 (99.5%)	846	16/16 (100%)	830/830 (100%)
Influenza A H3	1796	126/129 (97.7%)	1660/1667 (99.6%)	848	22/28 (78.6%)	819/820 (99.9%)
Influenza B	1796	38/39 (97.4%)	1748/1757 (99.5%)	851	13/15 (86.7%)	832/836 (99.5%)
Mycoplasma pneumoniae	1796	16/16 (100%)	1766/1780 (99.2%)	851	2/2 (100%)	845/849 (99.5%)
Parainfluenza Virus 1	1796	1/1 (100%)	1795/1795 (100%)	851	14/16 (87.5%)	835/835 (100%)
Parainfluenza Virus 2	1796	8/10 (80%)	1784/1786 (99.9%)	851	2/2 (100%)	848/849 (99.9%)
Parainfluenza Virus 3	1796	73/75 (97.3%)	1705/1721 (99.1%)	851	45/47 (95.7%)	803/804 (99.9%)
Parainfluenza Virus 4	1796	8/9 (88.9%)	1786/1787 (99.9%)	851	8/9 (88.9%)	841/842 (99.9%)
Respiratory Syncytial Virus	1796	173/176 (98.3%)	1605/1620 (99.1%)	851	27/28 (96.4%)	817/823 (99.3%)

+No positive results were identified by the comparator method.
